# The Effectiveness of Dance Therapy as an Adjunct to Rehabilitation of Adults With a Physical Disability

**DOI:** 10.3389/fpsyg.2020.01963

**Published:** 2020-08-26

**Authors:** Bonnie Swaine, Frédérique Poncet, Brigitte Lachance, Chloé Proulx-Goulet, Vicky Bergeron, Élodie Brousse, Julie Lamoureux, Patricia McKinley

**Affiliations:** ^1^School of Rehabilitation, Faculty of Medicine, Université de Montréal, Montréal, QC, Canada; ^2^Centre for Interdisciplinary Research in Rehabilitation of Greater Montreal (CRIR), Montréal, QC, Canada; ^3^Institut Universitaire sur la Réadaptation en Déficience Physique de Montréal (IURDPM), CIUSSS du Centre-Sud-de-l’Île-de-Montréal, Montréal, QC, Canada; ^4^Laboratory for Adult Development and Cognitive Aging, Department of Psychology, Concordia University, Montréal, QC, Canada; ^5^School of Physical and Occupational Therapy, Faculty of Medicine, McGill University, Montréal, QC, Canada

**Keywords:** dance therapy, physical disability, effectiveness, rehabilitation, adults

## Abstract

**Background/Objective:** To determine the added benefit on participants’ mobility and participation of a 12-week dance therapy (DT) intervention combined with usual physical rehabilitation for adults with varied physical disabilities. Their appreciation of DT was also explored.

**Methods:** We conducted a quasi-experimental study pre–post test with a nonequivalent control group and repeated measurements pre, post, and at a 3-month follow-up.

**Results:** Although participants in both groups significantly improved over time (at 12 weeks and at follow-up) compared to baseline on mobility (timed up and go, TUG) and participation (e.g., Life-H scores and number of leisure activities), treatment effect analysis using propensity score matching showed no significant treatment effect of DT. The TUG scores showed the best promise of a treatment effect. DT participants’ Flow State Scale scores significantly improved (*p* < 0.01) for 5/9 dimensions of flow (being in control, loss of self-consciousness), and they all recommended DT.

**Conclusion:** This study failed to demonstrate an added benefit of the DT intervention in improving participants’ mobility and participation. Overwhelmingly, favorable participants’ opinions about the intervention support its potential impact.

## Introduction

Alternative treatment modalities are gaining popularity in rehabilitation including dance therapy (DT). DT improves aspects of physical, cognitive, and psychological function in specific homogenous groups of persons with stroke (Patterson et al., 2018) and Parkinson’s disease (McKinley et al., 2008; de Dreu et al., 2015) and among healthy individuals (O’Toole et al., 2015). Fewer studies have examined the impact of DT at the social level (i.e., participation) and on making lifestyle changes. Activity level increased in sedentary elderly (Kattenstroth et al., 2013) and among people with multiple sclerosis (MS) during and after a DT intervention (Mandelbaum et al., 2016). Participation in a community dance program appears to have increased the repertoire of activities in a child with cerebral palsy (López-Ortiz et al., 2012) and in elderly women (Nadasen, 2008). Finally, increased frequency of participation in activities among healthy seniors (O’Toole et al., 2015) and in social activities among patients with Parkinson’s disease (Foster et al., 2013) is noted.

Indeed, the literature about the impacts of DT is growing. Most studies are, however, conducted in community settings, often with an inactive control group; only two studies involve heterogeneous groups of participants with various diseases, although the results seem encouraging (Selman et al., 2012; Krampe et al., 2014). Only one study assessed the added benefit of DT compared to traditional rehabilitation for adults with chronic back pain (Okafor et al., 2012) and found that the aerobic dance group reported significant effects in pain intensity, functional disability, and quality of life. We found no studies regarding the effect of DT as an adjunct to physical rehabilitation.

To address the gaps in the literature about using DT among heterogeneous rehabilitation service users, we explored the effect of DT on functional mobility and social participation among DT participants receiving active rehabilitation treatment compared to that of a control group receiving only traditional rehabilitation. Specifically, we sought to determine the added benefit of DT to traditional rehabilitation and whether DT enables the maintenance of participants’ improvements in participation. Given the goals of the DT intervention (described below), we hypothesized that participants would improve their functional mobility and consequently be more physically active following the intervention, allowing them to participate more fully in community activities and thus improve their participation. We also deemed it important to explore participants’ perceptions regarding their appreciation of the intervention.

## Materials and Methods

### Study Design

We conducted a quasi-experimental study pre–post design with a nonequivalent control group and repeated measurements at baseline, the end of the DT intervention, and 3 months later. A randomized clinical trial was not possible since the DT intervention had become standard care in 2009, with about 60 adults with various physical disabilities receiving the intervention per year since then.

### Setting

The study took place at the Lucie-Bruneau Rehabilitation Centre of the *Centre intégré universitaire de santé et de services sociaux (CIUSSS) du Centre-Sud-de-l’Île-de-Montréal* (Montréal, Québec, Canada), a facility providing interdisciplinary *outpatient* rehabilitation services to adults with various physical disabilities including acquired brain injury, degenerative diseases, and chronic pain.

### Intervention

The DT intervention is offered to groups of 10–20 rehabilitation clients and consists of 12 weekly sessions of 90 min (Lachance et al., 2018). Each session is divided into three parts: (1) warm-up (20 min); (2) exploration of a theme (50 min); and (3) relaxation (20 min). This DT is based on the theories of Laban Movement Analysis (Laban, 1947), dance improvisation and choreography, somatic education, group process, and rehabilitation principles such that the aim of the DT is not to succeed in performing a specific set of movements but rather to explore a diversity of movements through different movement themes such as time and space. The intervention is linked to the rehabilitation center’s mission aiming to facilitate social integration and participation.

Two rehabilitation clinicians (a physiotherapist, BL, and an occupational therapist, CP-G, each trained in dance) are the instructors providing the intervention. Sessions take place in a large well-lit room at the center furnished with chairs, floor mats, and pillows. A portable sound system provides the music.

### Participants

We recruited study participants the same way clinicians recruit rehabilitation clients for the DT intervention. Clinicians working within the clinical programs are informed about the DT group through meetings with the instructors and via posters about upcoming sessions. Clinicians speak about DT to patients meeting the eligibility criteria and then provide names of interested participants to the instructors. Similarly, clinicians provided names of interested participants to a research coordinator who then spoke to them about the study. About half of the people we spoke to were interested and enrolled in the DT and the study; exact numbers were impossible to obtain given the clinical context in which the study occurred. Some interested people sign up for the DT, but before a session begins, they might have progressed well in their rehabilitation enabling a return to work so their discharge from rehabilitation prevents them from attending the DT. Persons interested in the DT, but who were not interested in attending the session at the time of study enrollment, were invited to participate in the control group and could take part in a future DT session. Control group participants did not attend any DT intervention session during the study period. All participants received a variety of rehabilitation interventions from one or more disciplines (e.g., occupational therapy, social work, and speech therapy), the intensity, and frequency/duration being based on the individual’s needs during the 12-week period. Besides all receiving active rehabilitation on an outpatient basis at the rehabilitation center, eligible patients could follow verbal or visual cues, knew their physical limits in order to participate safely, were interested and motivated to improve their health by making positive changes in their practice of physical exercise and creative expression, and wanted to improve their functioning and autonomy in terms of balance, mobility, and confidence in their physical abilities. Excluded were people with significant behavioral problems.

All participants provided informed consent, and the project was approved by the Ethics Committee of the Centre for Interdisciplinary Research in Rehabilitation of Greater Montreal.

### Procedure

Participants were recruited over 5 sessions of DT between March 2014 and December 2015. Participants were assessed 4 times: at baseline occurring 3 weeks before the DT intervention (T0), during the week prior to the first class or during its first week (T1), during the last week of the intervention (T2), and 3 months later (T3). Persons independent from the DT group (e.g., graduate students) were trained during a half-day session to administer the assessment tools; they were not blind to group assignment since often evaluations occurred immediately following a DT class. Each participant was assessed individually in a distraction-free evaluation room.

### Outcome Measures

Participants in both groups were assessed using 3 tools chosen for their strong psychometric properties, their applicability with patients of varying diagnoses, and their links to the intervention objectives and center’s mission.

Our primary outcome measure (i.e., participation) was assessed, during a 30- to 60-min interview, using parts of the abridged version of the Assessment of Life Habits (LIFE H 3.0; Noreau et al., 2002; Lemmens et al., 2007). Although the tool measures 12 domains or life habit categories, we used it to assess participants’ level of participation with respect to 3 domains: moving around in their community, to being active in the community and leisure involvement. Qualitative data from interviews with DT participants in an earlier exploratory study (Lachance et al., 2013) indicated that these aspects were common and important goals for DT participants. The LIFE-H is valid for use in different populations (Poncet et al., 2018) and demonstrates good internal consistency and test–retest reliability (Labbe, 2000; Figueiredo et al., 2010).

The *Profil du Loisir* (Leisure profile) provided another measure of participation: participants’ involvement in leisure activities (Dutil et al., 2007) or the number of activities participants were involved in over the study. The tool demonstrated good inter-rater and test–retest reliability (Bier et al., 2009).

The Timed Up and Go (TUG) provided a measure of mobility, speed, and functionality (Podsiadlo and Richardson, 1991) and was assessed only with participants who could walk with or without an assistive device. The critical threshold for the TUG is ≥13.5 s to identify users at risk of falling. This test is done very quickly (<5 min.), and test–retest reliability estimates range from adequate to excellent according to the population studied. Inter-/intra-rater reliability is excellent (Shumway-Cook et al., 2000).

Only participants in the experimental group completed the Flow State Scale version 2 (FSS-2; Jackson and Eklund, 2002), a self-administered questionnaire that measures the concept of “flow” defined as a state in which the individual has an experience so pleasant and enjoyable that there is an increased desire to repeat it. The FSS-2 contains 9 dimensions: a challenge-skill balance, merging of action and awareness, having clear goals, unambiguous feedback, total concentration on the task, a sense of being in control, loss of self-awareness, loss of time awareness, and autotelic experience. Each dimension is represented by 4 questions (total score = 36), and responses are recorded using a scale ranging from (1) strongly disagree to (5) strongly agree. A total flow score is calculated by adding the subscores of the 9 dimensions, higher scores indicating a better experience. Its reliability is good, and the test takes less than 10 min to administer but must be completed within the hour following an activity (i.e., the DT intervention). This test was thus administered only to the experimental group at T1 and T2.

Semi-structured interviews with participants in the experimental group at T2 enabled obtaining their opinions about the intervention: (1) What did you get from the DT in addition to your rehabilitation? (2) Would you recommend the DT to other patients? (3) Are there things you did not like in the dance intervention?

We also recorded the type of assistive walking device used by participants, and a variable was created and categorized to indicate progress from baseline in technical aids in a qualitative fashion: regression, maintenance, or progression.

### Analysis

Descriptive statistics (e.g., mean, standard deviation, median, and interquartile ranges) describe the participants’ characteristics at baseline. Since differences were found between groups with respect to some baseline measures, we used propensity score matching to control for those differences in the analyses described in detail below. The distribution of data was evaluated, and data were transformed when necessary to meet the assumptions of each statistical test.

A treatment effect analysis, using propensity score matching, was performed to reduce the treatment assignment bias and mimic randomization. It estimates the average treatment effect (ATE) and average treatment effect on the treated (ATET) from observational data (Lunceford and Davidian, 2004). Specifically, propensity score matching estimators impute the missing potential outcome for each participant by using an average of the outcomes of similar participants that receive another treatment level. Similarity between participants (based on estimated treatment probabilities, known as propensity scores) is computed from baseline variables describing our participants. Treatment effect is computed by taking the average of the difference between the observed and potential outcomes for each participant. Simply stated, the analysis used creates a sample of units that received the treatment (DT group) that is comparable on all observed covariates to a sample of units that did not receive the treatment (control group). In other words, propensity matching controls for potential biases by making the groups receiving the dance intervention (DT) and only usual rehabilitation (not-treatment or control group) comparable with respect to the baseline variables.

These analyses were used to determine effect treatment on functional and participation scores. The treatment effect analysis using propensity score matching assumes overlap, i.e., everyone has a positive probability of receiving treatment. Because there were no participants with para/tetraplegia that participated in the DT group in this study, some participants (*n* = 8) do not have a positive probability of being assigned to the DT group and therefore were excluded from all of the treatment effect analyses.

The general linear model for repeated measures assessed change among DT participants for FSS-2 scores pre–post intervention. The level of significance was set at *p* < 0.05 for all statistical tests. All analyses were conducted using STATA 15.0 (StataCorp, TX, United States).

A qualitative content analysis was conducted with the responses to questions posed at T2 during interviews with the DT intervention participants.

## Results

### Participants

**FIGURE 1:**
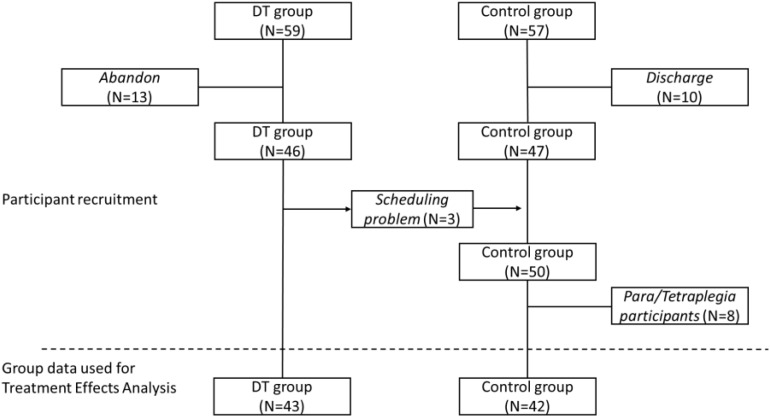
Recruitment flowchart for the dance therapy (DT) and control groups.

The DT and control groups were composed of 43 and 50 (42 for the ATE analysis) participants, respectively. Only data from participants who attended at least 9 of the 12 sessions were included. Initially, 59 persons participated in the DT group and 57 participants were in the control group. However, 13 participants in the DT group stopped participating (for reasons related to illness or transportation/scheduling difficulties), while 10 participants in the control group dropped out before the end of the study for a variety of reasons, but mostly because they were discharged from rehabilitation. Three participants from the experimental group transferred into the control group due to availability/scheduling issues before the DT session began (see Figure 1). This resulted in a dropout rate of 17.5% in the control group and 27.1% in the DT group; these rates were not significantly different between the two groups (χ^2^_1df_ = 1.528, *p* = 0.216).

**FIGURE 2:**
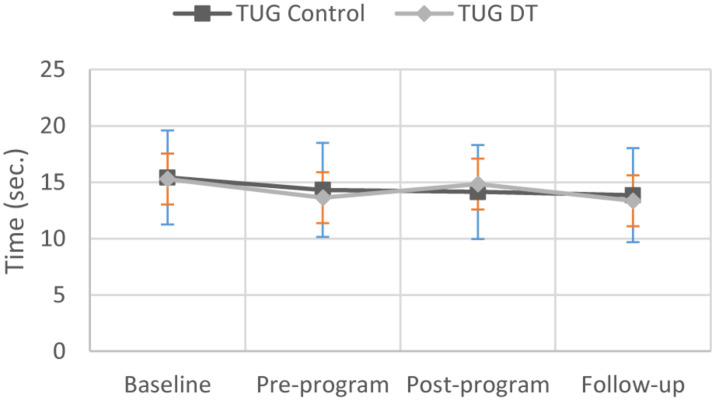
Changes in Timed Up and Go test scores for participants in the control and dance therapy intervention groups.

**FIGURE 3:**
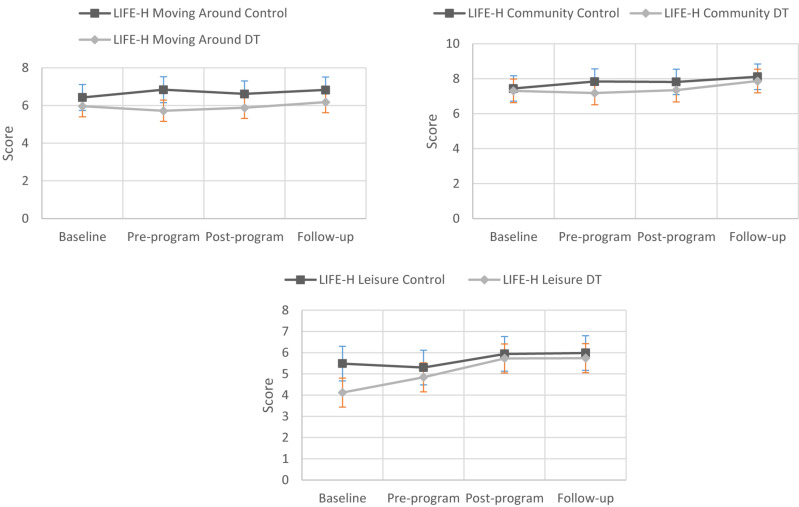
Changes in LIFE-H scores for participants in the control and dance therapy intervention groups.

**FIGURE 4:**
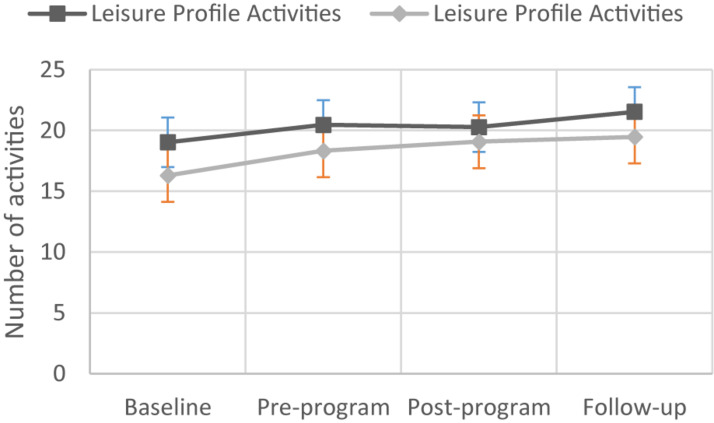
Changes in Leisure Profile Activities scores for participants in the control and dance therapy intervention groups.

Table 1 reports the baseline demographic and performance characteristics of participants. Most of the study participants had an acquired brain injury, 15 and 23 in the DT group and control group, respectively. Six people in the DT group had MS, while four people in the control group had the disease. Overall, 28.0% of participants had a degenerative condition, and this percentage was significantly greater (χ^2^_1df_ = 5.323, *p* = 0.021) in the DT group (39.5%) compared to the control group (18.0%). The majority of participants required an assistive walking device, 27 and 22 in the DT and control groups, respectively. With respect to the mean age (years ± SD), DT group participants (52.1 ± 13.6) were slightly (but not significantly) older (t_91df_ = 1.896, *p* = 0.061) than those in the control group (49.6 ± 14.4). Mean LIFE-H-Leisure score was significantly higher (*t*_91df_ = 2.205, *p* = 0.003) in the control group (5.22 ± 2.47) compared to the DT group (4.12 ± 2.30). See Figures 2–4 for changes in data for all variables for both groups.

**TABLE 1:** Descriptive statistics and comparability of groups at baseline (85 participants, 42 control, and 43 DT).

	DT	Control	Test	Sig.
Gender (% female)	55.8	42.9	χ^2^_1df_ = 2.098^a^	0.039
Age [average (SD)]	52.1 (13.6)	45.9 (14.4)	*t*_83df_ = 1.896^b^	0.061
Degenerative condition (% yes)	39.5	21.4	χ^2^_1df_ = 3.281^a^	0.070
Low function (% TUG > 13.5)	55.6	31.7	χ^2^_1df_ = 4.452^a^	0.035
TUG [average (SD)]	15.27 (6.70)	15.41 (6.70)	*z* = 1.221^c^	0.220
LIFE-H Moving around [average (SD)]	5.96 (1.78)	6.43 (2.26)	*t*_83df_ = 0.363^b^	0.718
LIFE-H Community [average (SD)]	7.31 (2.21)	7.44 (2.51)	*t*_83df_ = 0.265^b^	0.791
LIFE-H Leisure [average (SD)]	4.12 (2.30)	5.48 (2.51)	*t*_91df_ = 2.205^b^	0.003
Leisure Profile number of activities [average (SD)]	16.30 (7.06)	19.02 (6.82)	*z* = 1.863^c^	0.063

*Legend: SD: Standard deviation; TUG: Timed Up and Go; ^*a*^Chi-square test, ^*b*^Independent *t*-test, and ^*c*^Wilcoxon rank-sum test.*

Considering the differences at baseline on some clinical and performance variables, we used variables with a level of significance *p* < 0.1 when comparing groups at baseline (age, degenerative condition, low function at baseline, LIFE-H Leisure score at baseline, and number of activities on leisure profile) to compute propensity scores and perform the treatment effect estimations on the propensity score-matched results.

### Effect Measures

When considering the results on outcomes at the end of the 12-week period, there were no significant treatment effects of DT. Table 2 reports the ATEs and levels of significance for each outcome. Due to the propensity score matching, the N varies depending on the outcome variable analyzed.

**TABLE 2:** Results of treatment effect analysis using propensity score matching at the conclusion of the 12-week dance therapy program.

Outcome	N	ATE	SE	*Z*	Sig	95% CI
TUG	79	−1.802	2.34	−0.77	0.441	[−6.389;2.785]
LIFE-H Moving around	80	−0.633	0.523	−1.21	0.226	[−1.659;0.392]
LIFE-H Community	80	−0.408	0.526	−0.78	0.438	[−1.439;0.623]
LIFE-H Leisure	80	−0.402	0.665	−0.60	0.546	[−1.706;0.902]
Leisure Profile number of activities	79	1.519	1.804	0.84	0.400	[−2.016;5.054]

*N: Number of participants included in analysis, ATE: Average treatment effect, SE: Standard error of ATE, *z*: Statistic test, Sig: Significance of the statistic test (H0 is ATE = 0), 95% CI: 95% confidence interval.*

When considering the results on outcomes at the 3-month follow-up, TUG showed the best promise of a treatment effect, even if the level of significance is not reached (*p* = 0.052; see Table 3). The TUG improves an average of 2 s at the 3-month follow-up for the DT group compared to the control group.

**TABLE 3:** Results of treatment effect analysis using propensity score matching at follow-up (3 months post-conclusion of the 12-week program).

Outcome	N	ATE	SE	Z	Sig	95% CI
TUG	80	−2.179	1.123	−1.94	0.052	[−4.380;0.023]
LIFE-H Moving around	81	−0.168	0.553	−0.30	0.762	[−1.253;0.918]
LIFE-H Community	81	−0.800	0.761	−1.05	0.293	[−2.292;0.692]
LIFE-H Leisure	81	−0.878	0.657	−1.34	0.181	[−2.165;0.409]
Leisure Profile number of activities	81	−2.197	2.091	−1.05	0.293	[−6.296;1.901]

*N: Number of participants included in analysis, ATE: Average treatment effect, SE: Standard error of ATE, z: Statistic test, Sig: Significance of the statistic test (H0 is ATE = 0), 95% CI: 95% confidence interval.*

**FIGURE 5:**
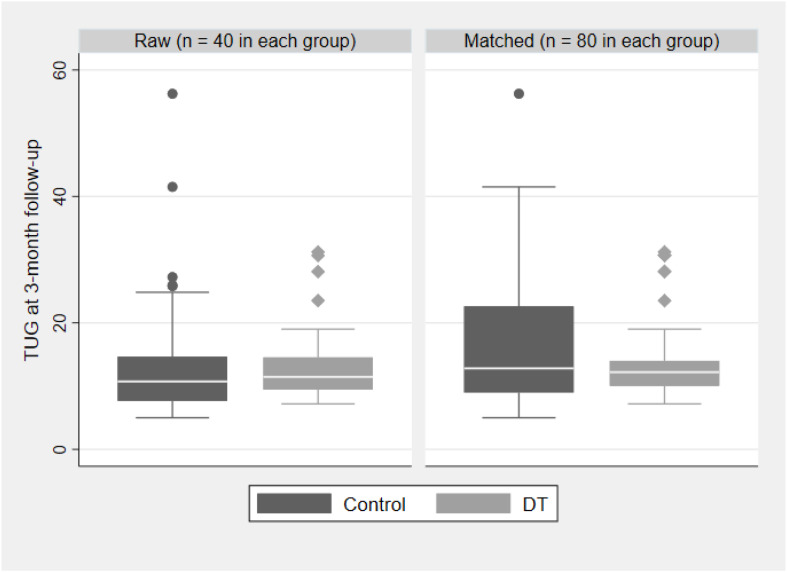
Propensity score matching for the Timed Up and Go scores for the control group versus the dance therapy group.

Figure 5 indicates that after propensity score matching, the control group shows a tendency for a higher TUG compared to the DT group. A *post hoc* power analysis suggests that to detect a minimum difference of 3 s between the groups at follow-up, we would have needed a minimum of 113 participants. Our final matched sample had 80 participants (40 from each group), and the raw difference between the DT and control group was about 2 s, indicating our study was underpowered for this outcome.

FSS-2 scores significantly improved among DT participants (*p* = 0.008 to 0.01) for 5/9 dimensions of flow experience between T1 and T2 (see Table 4): (1) “Merging of action and awareness,” (2) “Having clear goals,” (3) “A sense of being in control,” (4) “Loss of self-consciousness,” and (5) “Autotelic experience.” FFS-2 total scores significantly increased about 10 points between weeks 1 and 12 (*z* = 3.08, *p* = 0.002). In no model was the interaction between measurement time (week 1 and week 12) and gender significant, indicating that the evolution over time was not significantly different between men and women.

**TABLE 4:** Mean Flow State Scale scores for dance therapy participants during the first and last weeks of the 12-week session, controlling for gender.

Dimensions of flow	Week 1 mean score (+SD)	Week 12 mean score (+SD)	Test	Significance
Challenge-skill balance	15.1 (3.1)	15.4 (2.8)	*z* = 0.84	*p* = 0.401
Merging of action and awareness	13.0 (4.1)	14.5 (2.8)	*z* = 2.66	*p* = 0.008
Clear goals	13.3 (3.7)	14.4 (3.5)	*z* = 2.86	*p* = 0.004
Unambiguous feedback	14.1 (3.2)	14.7 (2.8)	*z* = 1.21	*p* = 0.228
Concentration on task at hand	15.9 (3.8)	16.5 (2.2)	*z* = 1.10	*p* = 0.269
Sense of control	13.6 (3.6)	14.7 (3.2)	*z* = 2.12	*p* = 0.034
Loss of self-consciousness	14.7 (4.8)	16.7 (4.0)	*z* = 2.66	*p* = 0.008
Transformation of time	13.7 (3.8)	15.0 (3.8)	*z* = 1.76	*p* = 0.079
Autotelic experience	16.8 (3.4)	18.3 (1.9)	*z* = 3.16	*p* = 0.002
Total score	130.2 (24.8)	140.2 (17.1)	*z* = 3.08	*p* = 0.002

*SD refers to standard deviation. N.B. Dimensions (and total score) in bold significantly improved over the 12-week intervention.*

Participants were unanimous (100%) in recommending DT (Table 5). Some of them (34.2%) reported elements of DT they did not like. However, responses varied widely depending on each person’s condition and preferences. For example, some participants felt that the period allowed for dancing was sometimes too short. Additional comments included the following: “*When you do DT, you stop thinking it’s therapy*”; “*The group was an opportunity for me to increase my balance and I got up my courage to go out dancing in a bar like I used to*”; and “*I can go to Jean-Talon market again, walk in a crowd*.”

**TABLE 5:** The 10 most frequent responses to the question: What has dance therapy provided you in addition to your rehabilitation?

Responses	Frequency (%)
It enabled me to relate to others	70.5%
I enjoyed myself	68.2%
I was able to move, improve my coordination, exercise	63.6%
I have more self-confidence	47.7%
My balance has improved	36.4%
I am more comfortable in space	36.4%
I feel better	31.8%
I was able to go beyond my limits	29.6%
I have a better body image	25.0%
I have higher self-esteem and an improved self-image	22.7%

## Discussion

The main objective was to explore the effect of DT on functional mobility and participation (particularly involvement in the community) of a heterogeneous group, receiving a 12-week, 90-min per week DT intervention in addition to their rehabilitation, compared to that of a control group receiving usual rehabilitation. Although participants in both groups improved after the 12-week assessment period and at 3-month follow-up compared to baseline, treatment effect analysis using propensity score matching showed no significant treatment effect of DT.

These results were unexpected; however, nonsignificant results such as these are not uncommon in DT research [see scoping review by Cherriere et al. (2019)]. For example, McGill et al. (2019) in a recent study failed to demonstrate significant effects of weekly ballet classes on gait variability or balance confidence among people with Parkinson’s disease. With regard to improvements in mobility (i.e., TUG scores), the present results are also consistent with the conclusion of a meta-analysis investigating the effects of dance on people with Parkinson’s disease (Shanahan et al., 2015). This review concluded that there was no evidence that dance is more effective than any other intervention in improving functional mobility. Based on our results, the TUG may be a promising tool for future investigations of the effect of DT on persons with a physical disability.

Others, however, have demonstrated significant improvements in the repertoire of activities of participants in a dance group (Nadasen, 2008; López-Ortiz et al., 2012). The frequency of participation in activities (mainly domestic) improved among a single (noncontrolled) group of people 50 years and over attending a creative dance program once a week for 6 weeks (O’Toole et al., 2015). Sabari et al. (2015) reported people with Parkinson’s disease participating in a community-based dance group for at least 6 months (at least once a week) being significantly more engaged in social activities compared to a nonparticipating group. In a randomized control trial, Foster et al. (2013) demonstrated that an intervention of 1 h, 2 times a week for 12 months of tango dance significantly improved participation in complex activities of daily living, in the recovery of activities lost since diagnosis and engagement in new activities compared to a control group.

It is, however, important to note the trend toward a treatment effect of DT at the 3-month follow-up based on the improvement in TUG scores of an average of 2 s. Huang et al. (2011) reported a minimally detectable change of 3.6 s in persons with Parkinson disease, so one might argue that an average change of 2 s may be clinically important for a heterogeneous sample of rehabilitation service users.

A secondary objective was to explore participants’ perception regarding their enjoyment during the dance intervention and overall appreciation. All participants stated they would recommend the DT intervention to others. Indeed, we did not assess how much or whether participants (in both groups) enjoyed their usual rehabilitation services (e.g., physiotherapy treatments). Anecdotal evidence suggests that participants of the DT intervention find the dance intervention more pleasing than regular rehabilitation. Participants’ opinions about DT in our study are very similar to those found in other studies where participants reported appreciating the interaction with others, enjoying themselves and thinking it is a good complement to traditional rehabilitation (Demers et al., 2015). Studies report how much participants enjoy DT and how it allowed them to improve their physical abilities and enabled them to move and to exercise and that they all recommend it to others (Krampe et al., 2010). Participants also report having a lot of fun participating (O’Toole et al., 2015).

The notion that dance is a pleasurable activity for participants is supported by the significant improvement for the experimental group in 5 of the 9 FSS-2 dimensions. Results indicate that DT participants were more involved, more in control, and more detached from the regard of others and had a better understanding of the goals of the dance activity at the end of intervention compared to the beginning. They were more focused, had fun, and gained a sense of well-being. Others found similar results without using the FFS-2 tool. For example, a qualitative study found that some participants among a group of elderly women reported that since they had been part of the dance group, they were no longer afraid of what others thought of them (Nadasen, 2008). Other participants expressed their great satisfaction with their dance group/intervention, reporting, for example, that they feel inspired and focused on what they can do doing their dance group (Sabari et al., 2015). These comments are consistent with the significant improvement demonstrated in our study in the dimensions of “sense of control” and “loss of self-consciousness.” Although the results of the FSS-2 demonstrate participants are more inclined to repeat the activity again, we were not able to measure whether participants intended to continue or continued dancing in the community following discharge from rehabilitation.

Several reasons might explain the nonsignificant results. First, they may be related to the intervention itself, the type of dance approach used, its duration (12 weeks), and frequency (90 min. once a week). An approach using improvisation and *individual* creativity may not be effective since the dance style used and the presence or absence of a partner appeared to influence the results in other studies (Hackney and Earhart, 2009, 2010). Dancing with a partner can facilitate the development of a social network and increase chances of greater social participation outside a dance program (Foster et al., 2013). While participants danced a few times in pairs during the DT intervention, most of the time they danced alone. Some suggest, however, that a partner is not necessary for a dance intervention in rehabilitation but that a person who is more severely affected might feel more comfortable and confident to experiment with more complex movements with a partner (Hackney and Earhart, 2010; Foster et al., 2013; Romenets et al., 2015).

With regard to the intervention duration and frequency, indeed, the studies cited above reported significant results with dance interventions of longer durations (e.g., 12 months) and more frequent (e.g., 2 sessions per week). However, a dance program provided 3 times a week to inactive healthy older adults was found no more effective than aerobic exercise training in improving in TUG scores, walking speed, and health-related quality of life (Esmail et al., 2019).

Another reason could relate to the choice of assessment tools used and outcome measures. Besides being a complex tool to administer, the LIFE-H may not be sensitive enough to detect subtle changes in participation and community involvement over time, as was observed by Poncet et al. (2018). It is possible that aspects of cognitive or psychological function improved differentially among the 2 groups, but this was not measured in the present study.

Despite using propensity score matching to reduce the treatment assignment bias, and mimic randomization, another reason for the absence of significant results could be due to the heterogeneity of participants. As in the study by Shanahan, Morris et al. (Shanahan et al., 2015), some participants had more advanced conditions than others that can decrease the effect of treatment on these patients since improvements are more difficult to achieve. Duncan and Earhart also linked the lack of effect of their intervention to the heterogeneity of their participants (Duncan and Earhart, 2012). Ideally, conducting a randomized control trial would have addressed some of the issues related to the heterogeneity of our sample; however, the clinical context of our research did not allow the use of this type of design. Indeed, the control group may not have been comparable to the experimental group on other variables not measured in this study: the control group participants were people who did not wish to participate in the DT program at the time of the study, which could cause a selection bias. In other words, it is possible that persons in the experimental group had more severe disabilities or limitations on their activities, making them more likely to agree to participate in DT in addition to their rehabilitation, thinking that it would be beneficial for them. Differential response bias could have existed with DT participants unconsciously providing more favorable responses than those from the control group. Again, propensity score matching attempted to reduce these biases.

The use of repeated measurements and the standardized training of evaluators strengthened our study design. Having been conducted in a single center in a restricted geographic location, however, reduces the generalizability of the results. Due to the popularity and growing demand for the DT intervention, 2 90-min, 12-week sessions are currently offered to outpatient rehabilitation clients at the center. However, in the current context of budgetary constraints, future research must address how best to administer (optimal duration and frequency) DT interventions like the one under study as well as the challenges related to choosing appropriate outcome measures with the potential to capture the impact/essence of this multimodal activity. Exploring the use of alternative and ethically acceptable study designs such as single-case experimental design is warranted.

## Conclusion

A 12-week DT intervention combined with traditional rehabilitation failed to demonstrate an added benefit of the DT to usual rehabilitation in improving the participation and mobility among adults with various physical disabilities. The overwhelming favorable participants’ opinions about the DT intervention, however, support the potential impact of this intervention.

## Data Availability Statement

The raw data supporting the conclusions of this article will be made available by the authors, without undue reservation.

## Ethics Statement

The studies involving human participants were reviewed and approved by the Ethics Committee of the Centre for Interdisciplinary Research in Rehabilitation of Greater Montreal. The patients/participants provided their written informed consent to participate in this study.

## Author Contributions

BS, FP, BL, CP-G, and PM actively participated in the grant application (writing the protocol, choice of measurement tools, etc.) and in the interpretation of results. ÉB, FP, and VB coordinated the data collection. BL and CP-G offered the dance class. JL is a statistician. BS and FP were responsible for the statistical analyzes. All the authors participated in the writing of the manuscript.

## Conflict of Interest

The authors declare that the research was conducted in the absence of any commercial or financial relationships that could be construed as a potential conflict of interest.
